# In Vitro Susceptibility of *Ureaplasma urealyticum* and *Mycoplasma hominis* Isolates in Argentina

**DOI:** 10.1155/S1064744995000706

**Published:** 1995

**Authors:** Jorgelina Smayevsky, Silvia Relloso, Mariela Pundik, Alejandra Lanza, Gabriela Weltman, Carlos Bantar, Hebe Bianchini

**Affiliations:** 1 Department of Microbiology Centro de Educadón Médica e Investigaciones Clínicas Buenos Aires Argentina; 2 Laboratorio Bio-Ciencia Buenos Aires Argentina; 3 Laboratorio de Microbiologia Centro de Educadón Médica e Investigaciones Clínicas Billinghurst 2447 Buenos Aires 1425 Argentina

## Abstract

*Objective:* Our goal was to determine the in vitro susceptibility of *Ureaplasma urealyticum* and *Mycoplasma hominis* isolates to several antibiotics in Argentina.

*Methods:* Ninety-four strains of *U. urealyticum* and 18 strains of *M. hominis* isolated from cervical and urethral specimens were studied. Broth microdilution and agar dilution tests for minocycline, tetracycline, erythromycin, ciprofloxacin, and ofloxacin were performed.

*Results:* Both methods proved to be reliable and reproducible for *U. urealyticum* and *M. hominis*, with no major differences in results. The *U. urealyticurn* strains were inhibited by erythromycin at MICs ranging from ≤0.5 to >8 μ/ml. Ofloxacin showed the highest activity against this latter organism. No differences between tetracycline and minocycline MICs were observed with *U. urealyticum*. Two *M. hominis* strains displaying high MICs both to tetracycline and to minocycline were detected.

*Conclusions:* The emerging resistance of mycoplasmas to certain antibiotics emphasizes the need to undertake further surveillance studies on the clinical isolates of such organisms.


Infectious Diseases in Obstetrics and Gynecology 3:236–240 (1995)
© 1996 Wiley-Liss, Inc.

### In Vitro Susceptibility of *Ureaplasma urealyticum* and *Mycoplasma hominis* Isolates in Argentina

Jorgelina Smayevsky, Silvia Relloso, Mariela Pundik, Alejandra Lanza, Gabriela Weltman, Carlos Bantar, and Hebe Bianchini

*Department of Microbiology, Centro de Educación Médica e Investigaciones Clínicas (J.S., S.R., A.L., C.B., H.B.) and Laboratorio Bio-Ciencia (J.S., M.P., G.W.), Buenos Aires, Argentina*

### Abstract

***Objective:*** **Our goal was to determine the in vitro susceptibility of *Ureaplasma urealyticum* and *Mycoplasma hominis* isolates to several antibiotics in Argentina.**

***Methods:*** **Ninety-four strains of *U. urealyticum* and 18 strains of *M. hominis* isolated from cervical and urethral specimens were studied. Broth microdilution and agar dilution tests for minocycline, tetracycline, erythromycin, ciprofloxacin, and ofloxacin were performed.**

***Results:*** **Both methods proved to be reliable and reproducible for *U. urealyticum* and *M. hominis*, with no major differences in results. The *U. urealyticum* strains were inhibited by erythromycin at MICs ranging from ≤0.5 to >8 μg/ml. Ofloxacin showed the highest activity against this latter organism. No differences between tetracycline and minocycline MICs were observed with *U. urealyticum*. Two *M. hominis* strains displaying high MICs both to tetracycline and to minocycline were detected.**

***Conclusions:*** **The emerging resistance of mycoplasmas to certain antibiotics emphasizes the need to undertake further surveillance studies on the clinical isolates of such organisms.**
© 1996 Wiley-Liss, Inc.

Key words
Mycoplasmas, resistance, antibiotics

The antimicrobial susceptibility of the 2 most common genital mycoplasmas, *Ureaplasma urealyticum* and *Mycoplasma hominis,* is of interest because they have been recognized as pathogens in infections of the newborn,[1,2] arthritis,[2] and particularly sexually transmitted diseases (STDs) such as urethritis[3,4] and pelvic inflammatory disease.[3,4]

Since resistance to traditional drugs, such as tetracycline, used for treating these infections is widely known for both *U. urealyticum* and *M. hominis*,[5–9] alternative antimicrobial agents should be evaluated.

The purpose of the present study was to determine the susceptibility of clinical isolates of *U. urealyticum* and *M. hominis* to 5 antibiotics and compare the results obtained from agar dilution and broth microdilution tests.

### MATERIALS AND METHODS

Ninety-five isolates of *U. urealyticum* and 19 of *M. hominis* strains were tested. The *U. urealyticum* isolates included 1 reference strain (Mycotrin system) and 94 low-passage clinical isolates from the cervix, urethra, sperm, or first-catch urine. The *M. hominis* isolates included 1 *M. hominis* reference strain (PG 21) obtained from the U.S. National Institutes of Health and 18 low-passage clinical isolates from the cervix, urethra, or sperm. All clinical isolates were

Address correspondence/reprint requests to Jorgelina Smayevsky, Laboratorio de Microbiologia, Centro de Educación Médica e Investigaciones Clínicas, Billinghurst 2447, (1425) Buenos Aires, Argentina.

Received October 30, 1995
Accepted February 22, 1996

collected and identified from 1990 to 1992 and stored at −70°C until used.

*U. urealyticum* was identified by urease production in U9 broth[10] and typical colonies in *Ureaplasma* differential agar medium (A7).[10,11] *M. hominis* was identified by arginine hydrolysis, typical colonies in Hayflick modified-agar medium,[10] and growth inhibition by *M. hominis* antiserum (provided by David Rose).[12]

### Antimicrobial Agents

The reference standard powers for in vitro susceptibility testing of minocycline (Lederle Laboratories), tetracycline hydrochloride (Pfizer Laboratories, Groton, CT), erythromycin base (Leptit Laboratories), ciprofloxacin (Syntial Laboratories), and ofloxacin (Cilag Laboratories) were obtained from the manufacturers. All 5 were used for *U. urealyticum*, but erythromycin was excluded for *M. hominis* because of its natural resistance.

### Inoculum

Aliquots of stock culture were frozen at −70°C and 6 10-fold serial dilutions in U9 broth (pH 6.0) for ureaplasmas or arginine broth (pH 7.0)[13] for mycoplasmas were prepared in order to determine the number of color-changing units (ccu) present (0.1 ml of organism suspension/0.9 ml of broth). A ccu was designated as the reciprocal of the highest dilution at which growth was present, as evidenced by an alkaline shift in the phenol red pH indicator. The stock cultures were thawed to room temperature on the day of the assay and diluted in the appropriate broth without antibiotics to reach the $10^4$–$10^5$ ccu/ml range.[14] The inoculum size was checked at the time of the assay, and the organism suspensions were "activated" by 2-h incubation prior to inoculating the test media.

### Broth Microdilution Test

U9 broth (pH 6.0) and arginine broth (pH 7.0) were used for testing *U. urealyticum* and *M. hominis*, respectively.[14–16] Broth volumes of 100 μl were inoculated into each well of 96-well microtiter plates. A 100-μl antibiotic stock solution was added to the first well and serial 2-fold dilutions to the remaining wells in each row were inoculated with 100 μl of either organism inoculum until the final antibiotic concentration reached a range of 16–0.5 μg/ml. Separately, the wells without drugs containing serial 10-fold organism dilutions were used as a positive growth control, while the wells lacking organisms and drug were used as a sterility control. Microtiter plates were sealed and incubated for 48 h in an ambient atmosphere at 35°C. The plates were observed for evidence of growth (color change). The MIC was defined as the lowest antibiotic concentration at which the color remained unaltered (no growth), as compared with the control growth color change. All assays were performed twice on different days.

### Agar Dilution Test

Ureaplasma differential agar medium (A7) and Hayflick modified-agar medium (Hm) were used for testing *U. urealyticum* and *M. hominis*, respectively.[17] One milliliter of 2-fold serial antibiotic dilution was added to 9 ml of agar medium, to reach a final range of 16–0.5 μg/ml. For *U. urealyticum* and *M. hominis*, a $10^7$ ccu/ml was placed into each well of a Steers replicator and inoculated on Hm and A7 agar, reaching $10^4$ organism/spot. The plates without antibiotics were used as a positive growth control. All plates were incubated in an ambient atmosphere with 5% $CO_2$ for 48 h at 35°C and were viewed with a microscope at ×25 and ×100 magnifications. The MIC was defined as the lowest antimicrobial concentration in which no growth (no colony) was observed compared with the growth control plate.

### Susceptibility Test Control

*Staphylococcus aureus* ATCC 25923 was used as a control for potential interaction of antibiotics, medium components, and pH influence. This strain was tested by broth microdilution and agar dilution tests. The MICs of *S. aureus* ATCC 25923 were simultaneously performed in Muller-Hinton agar and Muller-Hinton broth by the microdilution method. This control was performed twice on 2 different days.

### RESULTS

The broth microdilution and agar dilution tests proved to be reliable and reproducible for *U. urealyticum* and *M. hominis*, with no major differences between the methods.

Table 1 shows the MIC results and resistance rates by both methods for 5 antibiotics against *U. urealyticum*. The erythromycin inhibition and MIC

TABLE 1. MICs of 5 antibiotics for 94 *U. urealyticum* strains by 2 different methods

| | Method[a] | | | | | | | |
|---|---|---|---|---|---|---|---|---|
| | Broth microdilution | | | | Agar dilution | | | |
| Antibiotic | $MIC_{50}$ | $MIC_{90}$ | Range | % R | $MIC_{50}$ | $MIC_{90}$ | Range | % R |
| Minocycline | ≤0.5 | 1 | ≤0.50–>16 | 5 | ≤0.5 | 2 | ≤0.50–>16 | 4 |
| Tetracycline | ≤0.5 | 2 | ≤0.50–>16 | 5.5 | ≤0.5 | 2 | ≤0.50–>16 | 3.5 |
| Erythromycin | 2 | 4 | ≤0.50–>8 | 1 | 0.5 | 2 | ≤0.50–>8 | 1 |
| Ciprofloxacin | 2 | 2 | ≤0.50–>8 | 9.5 | 2 | 2 | ≤0.50–>8 | 9.5 |
| Ofloxacin | 1 | 1 | ≤0.50–2 | 0 | 0.5 | 1 | ≤0.50–2 | 0 |

[a]MIC and range are given as μg/ml. R, resistance.

TABLE 2. MICs of 4 antibiotics for 18 *M. hominis* strains by 2 different methods

| | Method[a] | | | | | | | |
|---|---|---|---|---|---|---|---|---|
| | Broth microdilution | | | | Agar dilution | | | |
| Antibiotic | $MIC_{50}$ | $MIC_{90}$ | Range | % R | $MIC_{50}$ | $MIC_{90}$ | Range | % R |
| Minocycline | ≤0.5 | ≤0.5 | ≤0.50–>16 | 11 | ≤0.5 | ≤0.5 | ≤0.50–>16 | 11 |
| Tetracycline | ≤0.5 | ≤0.5 | ≤0.50–>16 | 11 | ≤0.5 | 2 | ≤0.50–>16 | 11 |
| Ciprofloxacin | ≤0.5 | 1 | ≤0.50–1 | 0 | ≤0.5 | 1 | ≤0.50–2 | 0 |
| Ofloxacin | ≤0.5 | ≤0.5 | ≤0.50–1 | 0 | ≤0.5 | 2 | ≤0.50–2 | 0 |

[a]MIC and range are given as μg/ml. R, resistance.

50% and 90% were slightly higher by the broth microdilution vs. the agar dilution test. Only 1 erythromycin-resistant strain was detected. Both methods yielded similar MIC 50%, MIC 90%, and resistance rates for all antibiotics, except that 1 strain showed resistance to minocycline by the broth microdilution test, but not by the agar dilution test. Ofloxacin was the most active antibiotic tested against *U. urealyticum*, whereas ciprofloxacin was somewhat less active (9.5% resistance).

The MICs and resistance rates for 4 antibiotics against *M. hominis* are given in Table 2. By both methods, 2 strains displayed high minocycline or tetracycline MICs, i.e., >16 μg/ml, 11% resistance. No differences between ofloxacin and ciprofloxacin MICs were observed, even for strains showing either high or low tetracycline MICs.

No major differences were found in the MICs of the 5 antibiotics against *S. aureus* ATCC 25293 control in U9 broth, A7 agar, or Hayflick agar. The MIC values were within the acceptable range established for Muller-Hinton broth and Muller-Hinton agar.[18]

### DISCUSSION

The wide spectrum of mycoplasma susceptibility tests so far described has been restricted to differences in technique, inoculum size, pH media, incubation conditions, and endpoint criteria.[15,16,19] The medium used for *U. urealyticum* and *M. hominis* is complex, containing 10–20% serum, which makes the MIC values more difficult to compare with those from other bacteria that grow in simple medium. It has been speculated that the complex media and conditions necessary for optimal culture and susceptibility testing of mycoplasmas may exert some effect on the activity or stability of various antimicrobial agents.[9] Robertson et al.[15] failed to find any apparent binding effect when testing the susceptibility of *U. urealyticum* strains against tetracycline, erythromycin, and rosaramicin, but the expected decrease in macrolide activity at pH 6.0 was observed.

Since mycoplasmas exhibit no turbidity as evidence of growth and both *M. hominis* and *U. urealyticum* die soon after reaching peak growth, the preparation of suitable inocula is more difficult than with common bacteria. Using inocula higher than recommended, the MICs frequently were raised over 2-fold.[14,17] Until standardized methods to test mycoplasma susceptibility are available, these inconsistencies will continue.

Comparing the results obtained with broth microdilution and agar dilution tests, we found no significant differences, as reported by Cummings and McCormack.[5]

In agreement with Robertson et al.,[15] the broth microdilution test seemed a simple and sensitive growth indicator.

Kenny et al.[17] contended that the agar dilution method was more useful, as it was able to detect mixtures of susceptible and resistant mycoplasmas as well as spontaneous resistant mutants. Furthermore, the agar dilution test affords the advantage of testing many strains at one time.

The prevalence of *U. urealyticum* strains displaying high MICs to erythromycin found in this study was lower than that described by other authors.[15,16] However, Waites et al.[9] reported a relatively narrow MIC range (0.25–4 μg/l) when testing *U. urealyticum* erythromycin resistance.

The 6-fluoroquinolones offer a useful alternative for the treatment of infections caused by gonococci and other genital pathogens that are resistant to penicillin and tetracycline. These quinolones are remarkable for being able to inhibit both *M. hominis* and *U. urealyticum* at MIC levels attainable in tissue. In our study, ofloxacin proved more active than ciprofloxacin against *U. urealyticum*, as reported by Kenny et al.[17] Likewise, we failed to detect any *M. hominis* strains that were resistant to either ciprofloxacin or ofloxacin, which was in agreement with other authors.[9,17,19]

Cummings and McCormack[5] reported that *M. hominis* isolates in the northeastern United States have become more resistant to tetracycline and minocycline during the past decade, but data available in Argentina are insufficient to anticipate a similar trend.

The low levels of clinical resistance obviously conflict with the outcome of therapy predicted from MIC or serum levels. Since few *U. urealyticum* and *M. hominis* infections are septicemias, the achievable serum drug level may not reflect its concentration at the actual site of infection. Therefore, serum levels exceeding the MIC may be required for a bacteriologic cure.

In conclusion, the emerging resistance of mycoplasmas to certain antibiotics emphasizes the need to undertake further surveillance studies on the clinical isolates of such organisms. Furthermore, in addition to in vitro susceptibility and pharmacokinetic studies, clinical trials including suitable antibiotics to be used in mycoplasmal or unreaplasmal infections are essential before extrapolating in vitro findings to human treatment.

### REFERENCES

1. Cassell GH, Crouse DT, Waites KB, Rudd PT, Davis JK: Does *Ureaplasma urealyticum* cause respiratory disease in newborns? Pediatr Infect Dis J 7:535–541, 1988.
2. Cassell GH, Waites KB, Crouse DT, et al.: Association of *Ureaplasma urealyticum* infections of the lower respiratory tract with chronic lung disease and death in very low birthweight infants. Lancet 2:240–245, 1988.
3. Cassell GH, Davis JK, Waites KB, Rudd PT, Talkington D, Horowitz S: Pathogenesis and significance of urogenital mycoplasmal infections. Adv Med Biol 24:93–115, 1987.
4. Taylor Robinson D, Al McCormack W: Mycoplasmas in human genitourinary infections. In Tully JG, Whitcomb RF (eds): The Mycoplasmas. Vol II. New York: Academic Press, pp 307–366, 1979.
5. Cummings MC, McCormack WM: Increase in resistance of *Mycoplasma hominis* to tetracyclines. Antimicrob Agents Chemother 34:2297–2299, 1990.
6. Evans RT, Taylor Robinson D: The incidence of tetracycline resistant strains of *Ureaplasma urealyticum*. J Antimicrob Chemother 4:57–63, 1978.
7. Roberts MC, Kenny GE: TetM tetracycline resistant determinants in *Ureaplasma urealyticum*. Pediatr Infect Dis J 5S:338–340, 1986.
8. Taylor Robinson D, Furr PM: Clinical antibiotic resistance of *Ureaplasma urealyticum*. Pediatr Infect Dis J 5S:335–337, 1986.
9. Waites KB, Duffy LB, Schmid T, Crabb D, Pate, Cassell GH: In vitro susceptibilities of *Mycoplasma pneumoniae, Mycoplasma hominis* and *Ureaplasma urealyticum* to sparfloxacin and PD 127391. Antimicrob Agents Chemother 35:1181–1185, 1991.
10. Taylor Robinson D: Mycoplasmas and mixed infections of the human male urogenital tract and their possible complications. In Razin S, Basile MF (eds): The Mycoplasmas. Vol IV. London: Academic Press, pp 27–63, 1985.
11. Shepard MC, Lunceford CD: Differential agar medium (A7) for identification of *Ureaplasma urealyticum* (human T mycoplasmas) in primary cultures of clinical material. J Clin Microbiol 3:613–625, 1976.
12. Wallace AC: Growth inhibitions test. In Razin S, Tully JG (eds): Methods in Mycoplasmology. Vol I. New York: Academic Press, pp 405–410, 1983.
13. Shepard MC: Culture media for ureaplasmas. In Razin S, Tully JG (eds): Methods in Mycoplasmology. Vol. I. New York: Academic Press, pp 137–146, 1983.
14. Senterfit L: Antibiotic sensitivity testing of mycoplasmas. In Tully JG, Razin S (eds): Methods in Mycoplasmology. Vol II. New York: Academic Press, pp 397–401, 1983.
15. Robertson JA, Coppola JE, Heisler OR: Standardized method for determining antimicrobial susceptibility of strains of *Ureaplasma urealyticum* and their response to tetracycline, erythromycin and rosaramicin. Antimicrob Agents Chemother 20:53–58, 1981.

16. Waites KB, Cassell GH, Canupp KC, Fernández PB: In vitro susceptibilities of mycoplasmas and ureaplasmas to new macrolides and aryl-fluoroquinolones. Antimicrob Agents Chemother 32:1500–1502, 1988.
17. Kenny GE, Hooton TM, Roberts MC, Cartwright FD, Hoyt J: Susceptibilities of genital mycoplasmas to the newer quinolones as determined by the agar dilution method. Antimicrob Agents Chemother 33:103–107, 1989.
18. Thornsberry C, Anhalt J, Barry AL, et al. (eds): Method for dilution antimicrobial susceptibility tests for bacteria that grow aerobically: Approved standard. Vilanova, PA: National Committee for Clinical Laboratory Standards, M7-A, 1985.
19. Kenny G, Cartwright FD: Susceptibilities of *Mycoplasma hominis* and *Ureaplasma urealyticum* to two new quinolones, sparfloxacin and WIN 57273. Antimicrob Agents Chemother 35:151–156, 1991.
